# Retinal Degeneration in MPS-IIIA Mouse Model

**DOI:** 10.3389/fcell.2020.00132

**Published:** 2020-03-04

**Authors:** Daniela Intartaglia, Giuliana Giamundo, Elena Marrocco, Veronica Maffia, Francesco Giuseppe Salierno, Edoardo Nusco, Alessandro Fraldi, Ivan Conte, Nicolina Cristina Sorrentino

**Affiliations:** ^1^Telethon Institute of Genetics and Medicine, Pozzuoli, Italy; ^2^Department of Translational Medicine, University of Naples Federico II, Naples, Italy; ^3^Department of Biology, University of Naples Federico II, Naples, Italy

**Keywords:** retina, MPS-IIIA, lysosomal storage disease, autophagy, Sanfilippo A syndrome

## Abstract

Mucopolysaccharidosis type IIIA (MPS-IIIA, Sanfilippo A) is one of the most severe lysosomal storage disorder (LSD) caused by the inherited deficiency of sulfamidase, a lysosomal sulfatase enzyme involved in the stepwise degradation of heparan sulfates (HS). MPS-IIIA patients show multisystemic problems, including a strong impairment of central nervous system (CNS), mild somatic involvement, and ocular manifestations that result in significant visual impairment. Despite the CNS and somatic pathology have been well characterized, studies on visual system and function remain partially explored. Here, we characterized the retina morphology and functionality in MPS-IIIA mouse model and analyzed how the SGSH deficiency affects the autophagic flux. MPS-IIIA mice exhibited a progressive retinal dystrophy characterized by significant alterations in visual function. The photoreceptor degeneration was associated with HS accumulation and a block of autophagy pathway. These events caused a reactive microgliosis, and a development of apoptotic processes in MPS-IIIA mouse retina. Overall, this study provides the first phenotypic spectrum of retinal disorders in MPS-IIIA and significantly contributes for diagnosis, counseling, and potential therapies development.

## Introduction

Mucopolysaccharidoses (MPSs) belong to a group of lysosomal storage disorders (LSDs) caused by the deficiency of lysosomal enzymes involved in the catabolism of complex polysaccharides, the glycosaminoglycans (GAGs). GAGs derived from the degradation products of proteoglycans and are localized on cell surfaces and in the intracellular matrices. MPSs are classified in seven families (I, II, III, IV, VI, VII, and IX) basing on wide diversity of phenotype, some are lethal within the first period of life and others have no significant impact on life duration. MPSs patients show chronic and progressive clinical symptoms with multisystem involvement including visceral, skeletal, muscular, neurological, and ocular manifestations. Ophthalmic problems are very common in MPS patients with variable severity and age of onset. They are associated with the accumulation of GAGs and comprise corneal clouding, glaucoma, atrophy of optic nerve, and retinal dysfunction. MPS-III disorders (Sanfilippo syndromes, MPS-IIIA–D) represent the most prevalent form of MPS due to the deficiency of four different lysosomal enzymes involved in the degradation of heparan sulfates (HSs). Patients of different MPS-III subtypes show a very severe neuropathology including behavioral problems (aggressiveness, hyperactivity, and sleep disturbance) and only mild somatic manifestations. Most of MPS-III patients show a severe retinopathy associated with pigmentary retinal degeneration associated to changes in electroretinogram, but few documented cases present with corneal opacity and optic nerve atrophy (Del Longo et al., 2018). Among Sanfilippo disorders, MPS-IIIA represents the most common form and the birth prevalence has been estimated as one per 100,000 live births (Muenzer, 2011). MPS-IIIA (MIM 252900) is caused by the deficiency of the sulfamidase, a lysosomal hydrolase (heparan N-sulfatase; SGSH) that catalyzes the stepwise degradation of HS. As a consequence, undegraded HS accumulates in the cells and tissues of affected patients causing a systemic cell impairment predominantly in the central nervous system (CNS). MPS-IIIA patients show a very rapid progression of neuronal and somatic pathology with also the ocular structures involvement. Neuropathology includes intellectual disability, learning abnormalities, sleep disturbance, and behavioral problems (Valstar et al., 2010). The main ocular manifestations reported in MPS-IIIA patients are represented by nerve atrophy, corneal changes, glaucoma, and retinopathy caused by the HS deposition (Fenzl et al., 2015; Wilkin et al., 2016). For this reason, an early diagnosis is crucial for a rapid intervention and amelioration of disease outcome. A well-characterized spontaneous mouse model of MPS-IIIA is actually used for understanding and designing therapeutic approaches for the treatment of MPS-IIIA disease (Bhattacharyya et al., 2001). This animal model quite resembles the pathophysiology together with behavioral abnormalities present in patients. MPS-IIIA mice show a systemic primary HS storage followed by accumulation of secondary materials like ganglioside, cholesterol, ubiquitinated proteins particularly evident in the CNS (Maccari et al., 2017). Recent evidences indicate the relevance of the autophagy impairment as one of pivotal mechanism involved in the development of secondary storage materials and in the progression of neurodegeneration. Indeed, the autophagy pathway plays a crucial role in removing misfolded protein-aggregates and damage organelles, promoting the cellular clearance from toxic materials in neurons and their survival (Settembre et al., 2008; Fraldi et al., 2016; Ntsapi et al., 2018). Our and other studies clearly demonstrated that the impairment of autophagy pathway in the CNS was associated with a development of inflammation processes (astroglia and microglia activation) and behavioral abnormalities that ultimately culminate with the death of MPS-IIIA mice (Sorrentino et al., 2013; Sambri et al., 2017). Moreover, no previous study in this animal model tried to assess the visual function and if it is affected by the storage pathology and the block of autophagy flux. Here, we deeply analyzed the retinal phenotype in MPS-IIIA mice and evaluated its possible correlation with ocular pathology observed in patients affected by this disorder. Interestingly, we have found a progressive degeneration of retinal photoreceptor population, associated with a strong vision dysfunction that culminated at 9 months of age, the advanced stage of the CNS pathology. Moreover, we also observed that retinal pathology was associated with an increase of HS accumulation and a block in lysosomal–autophagosomal fusion in MPS-IIIA mouse retina. These events cause the apoptosis-mediated cell death and inflammatory processes in the MPS-IIIA retina.

## Results

### Photoreceptor Cell Dysfunction in Retina of 3 Months Old MPS-IIIA Mice

**FIGURE 1:**
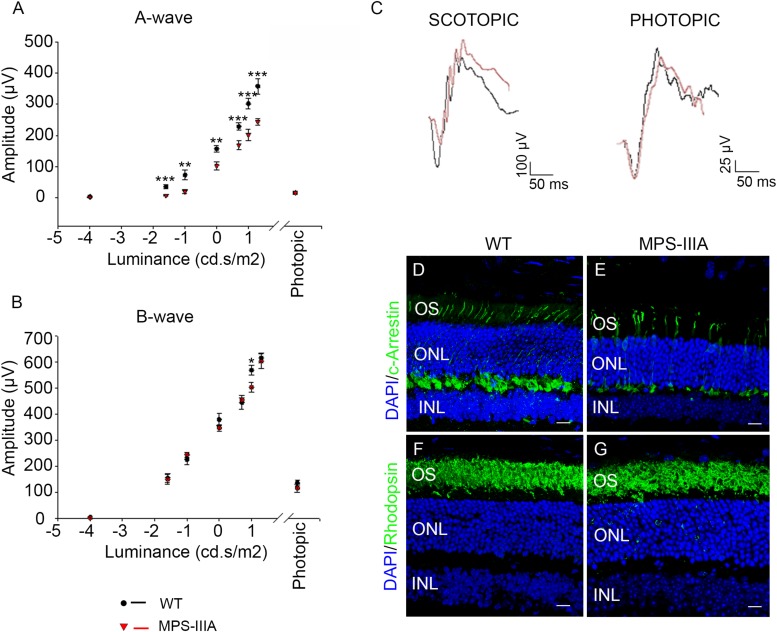
3 months old MPS-IIIA mice display initial signs of photoreceptors dysfunction. **(A,B)** Representative ERG (a- and b-wave), plotted as a function of stimulus intensity, from 3 months old WT (black circle) and MPS-IIIA (red triangle) mice. Error bars represent SEM. ***p-value ≤ 0.005, **p-value ≤ 0.01, *p-value ≤ 0.05 *t*-test. **(C)** Representative ERG (scotopic and photopic responses) traces of WT (black line) and MPS-IIIA (red line) mice at 3 months of age. Scotopic indicates ashes of 20.0 cd s/m^2^ light intensity; photopic indicates ashes of 20.0 cd s/m^2^ light intensity on a constant background illumination of 50 cd/m^2^. Representative images of retina cryosections immunostained with anti-c-Arrestin **(D,E)** and anti-Rhodopsin **(F,G)** antibodies from WT **(D,F)** and MPS-IIIA **(E,G)** mice at P90. Nuclei are counterstained with DAPI (blue). At least *n* = 6 mice per group. Scale bar 10 μm. OS, outer segment; ONL, outer nuclear layer; INL, inner nuclear layer.

In order to explore the retinal phenotypic alteration in MPS-IIIA disorders, we carried out a detailed functional and morphological analysis of male MPS-IIIA and age-matched wild type (WT) mice at different time points: 3-, 6-, 9 months of age according to progression of neuropathology. Retinal function was first evaluated by standard electroretinographic (ERG) recordings in mice at 3 months of age. Our results showed a decrease of a-wave amplitude with a relatively preserved b-wave amplitude in MPS-IIIA mice compared to age-matched WT mice (Figures 1A–C). Importantly, light intensities beyond -1.0 log cd s/m^2^ evoke responses originated in the mesopic range (i.e., by both rod and cone photoreceptors), therefore these data support the possibility that the recorded decrease of ERG was due to defects in both rod- and cone-derived components. We then examined whether the retinal functional degeneration in 3 months old MPS-IIIA mice is associated with morphological alteration of photoreceptor cells. This analysis was performed by measuring the density of both cones and rods in retinal sections labeled with anti-c-Arrestin and anti-Rhodopsin antibodies, which mark respectively cones and rods. Interestingly, we did not detect any significant alteration in rods and outer nuclear layer (ONL) thickness, but we observed a significant decrease in cone densities in MPS-IIIA mice compared to age-matched WT mice (Figures 1D–G). Overall, the above data suggest that at 3 months old, when the CNS pathology is not still evident, the MPS-IIIA mice show signs of photoreceptor dysfunction in spite of apparently normal morphology of rods.

### MPS-IIIA Mice Display a Progressive Retinal Dystrophy

**FIGURE 2:**
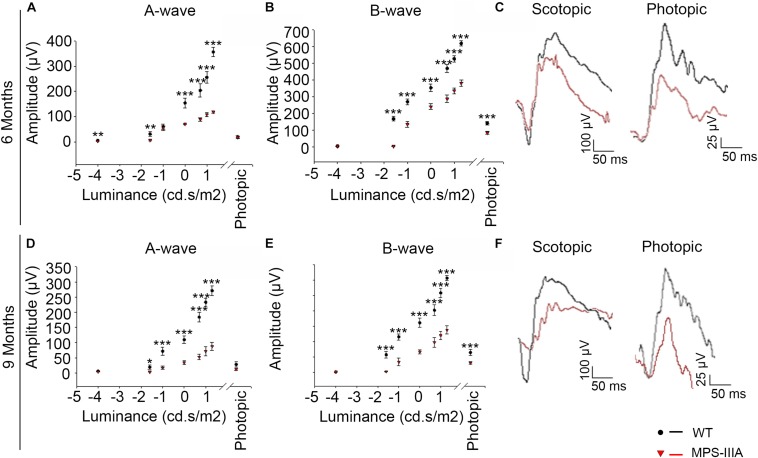
MPS-IIIA mice show a progressive functional photoreceptor deficiency. **(A,B)** Representative ERG (a- and b-wave), plotted as a function of stimulus intensity, from 6 months old WT (black circle) and MPS-IIIA (red triangle) mice. Error bars represent SEM. ***p-value ≤ 0.005, **p-value ≤ 0.01 *t*-test. **(C)** Representative ERG (scotopic and photopic responses) traces of WT (black line) and MPS-IIIA (red line) mice at 6 months of age. Scotopic indicates ashes of 20.0 cd s/m^2^ light intensity; photopic indicates ashes of 20.0 cd s/m^2^ light intensity on a constant background illumination of 50 cd/m^2^. **(D,E)** Representative ERG (a- and b-wave), plotted as a function of stimulus intensity, from 9 months old WT (black circle) and MPS-IIIA (red triangle) mice. Error bars represent SEM. ***p-value ≤ 0.005, *p-value ≤ 0.05 *t*-test. **(F)** Representative ERG (scotopic and photopic responses) traces of WT (black line) and MPS-IIIA (red line) mice at 9 months of age. Scotopic indicates ashes of 20.0 cd s/m^2^ light intensity; photopic indicates ashes of 20.0 cd s/m^2^ light intensity on a constant background illumination of 50 cd/m^2^.

**FIGURE 3:**
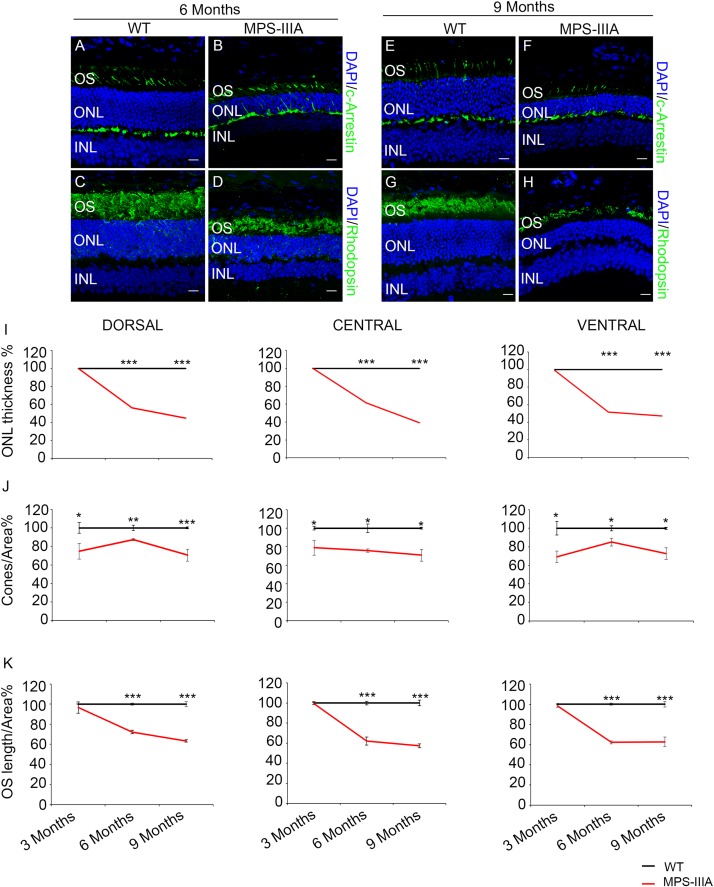
*SGSH* deletion results in progressive morphological photoreceptors defects. Representative images of retina cryosections immunostained with anti-c-Arrestin **(A,B,E,F)** and anti-Rhodopsin **(C,D,G,H)** antibodies from WT **(A,C,E,G)** and MPS-IIIA **(B,D,F,H)** mice at 6 months **(A–D)** and 9 months **(E–H)** of age. Nuclei are counterstained with DAPI (blue). At least *n* = 6 mice per group. Scale bar 10 μm. OS, outer segment; ONL, outer nuclear layer; INL, inner nuclear layer. **(I)** Graphs show the percentage of ONL thickness from the retina of WT and MPS-IIIA mice at 3-, 6-, and 9 months of age. Note that from 6 months of age onward, there are significant differences in the percentage of ONL layers number and thickness in each analyzed retina region (dorsal, central, and ventral) between WT and MPS-IIIA mice. Error bars represent SEM. ***p-value ≤ 0.005 *t*-test. **(J)** Graphs show cone percentage (cones/area) from the retina of WT and MPS-IIIA mice at 3-, 6-, and 9 months of age. From 3 months of age onward, there are significant differences in the percentage of cones number in each analyzed retina region (dorsal, central, and ventral) between WT and MPS-IIIA mice up to a greater than 30% reduction in MPS-IIIA mice. Error bars represent SEM. ***p-value ≤ 0.005, **p-value ≤ 0.01, *p-value ≤ 0.05 *t*-test. **(K)** Graphs show the reduction of the percentage of OS length from 6 months of age onward from each analyzed retina region (dorsal, central, and ventral) of WT and MPS-IIIA mice. Error bars represent SEM. ***p-value ≤ 0.005 *t*-test.

Usually, the earliest clinical symptom of retinal degeneration is an initial dysfunctional photoreceptor system. Subsequent degeneration of rods and cones leads to a complete loss of the visual field. To further explore the phenotypic consequences in the retina of MPS-IIIA, we expanded our morphological and functional analyses at later ages, focusing on 6- and 9 months old animals, in which pathological signs of neurodegeneration in the CNS are evident (Bhaumik et al., 1999; Sorrentino et al., 2013). Notably, the photoreceptors response, as assessed by ERG analysis, showed a progressive reduction, respectively, at 6- and 9 months, which reaches a 50% amplitude reduction in 9 months old MPS-IIIA mice respect to age-matched WT control mice. This reduction was also observed in b-wave responses (Figures 2A–F). According with a progressive and finally severe deficiency of photoreceptor function observed from the age of 3 months onward, MPS-IIIA mice showed a gradual loss of cones at these same stages (Figures 3A–K). Notably, 6 months old MPS-IIIA mice showed approximately a 20% decrease of cone density compared to age-matched littermates WT controls. Significantly, these alterations were associated with deficiencies in rod density and a significant decrease in ONL thickness (Figures 3A–D,I,K). Moreover, 9 months old MPS-IIIA mice showed approximately a 60% of rods density reduction and a strong reduction in ONL thickness, compared to age-matched WT control mice (Figures 3E–I,K). Overall, these data indicate that MPS-IIIA mice show a gradual retinal dystrophy phenotype according to the CNS pathology progression.

### Photoreceptor Degeneration Is Associated With Inflammation and Apoptotic Events in MPS-IIIA Mice

**FIGURE 4:**
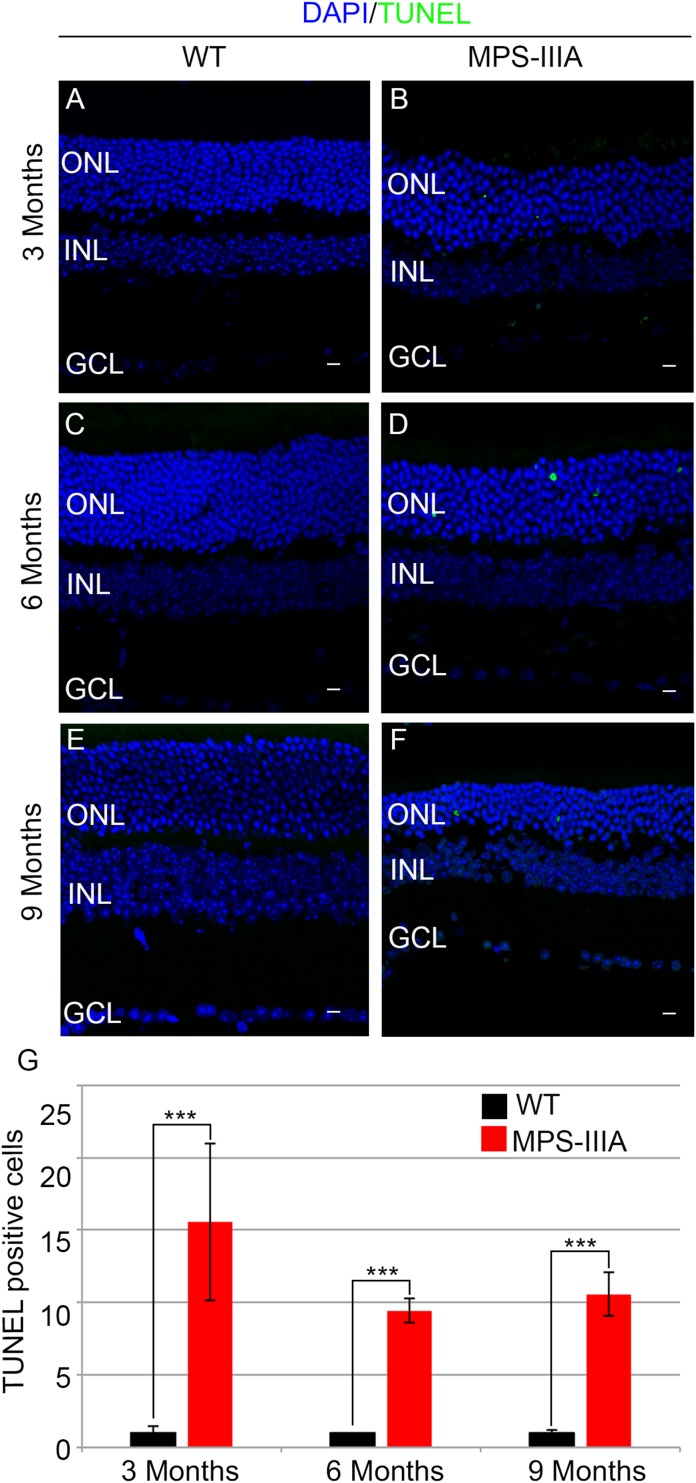
MPS-IIIA mice show an increase in retina cell death. Representative images of WT **(A,C,E)** and MPS-IIIA **(B,D,F)** retinas, at 3 months **(A,B)**, 6 months **(C,D)**, and 9 months **(E,F)** of age stained with TUNEL-fluorescein. Nuclei are counterstained with DAPI (blue). At least *n* = 6 mice per group. Scale bar 10 μm. ONL, outer nuclear layer; INL, inner nuclear layer; GCL, ganglion cell layer. **(G)** Graph shows the number of TUNEL positive cells from the retina of WT and MPS-IIIA mice at 3-, 6-, and 9 months of age. Error bars represent SEM. ***p-value ≤ 0.005 *t*-test.

**FIGURE 5:**
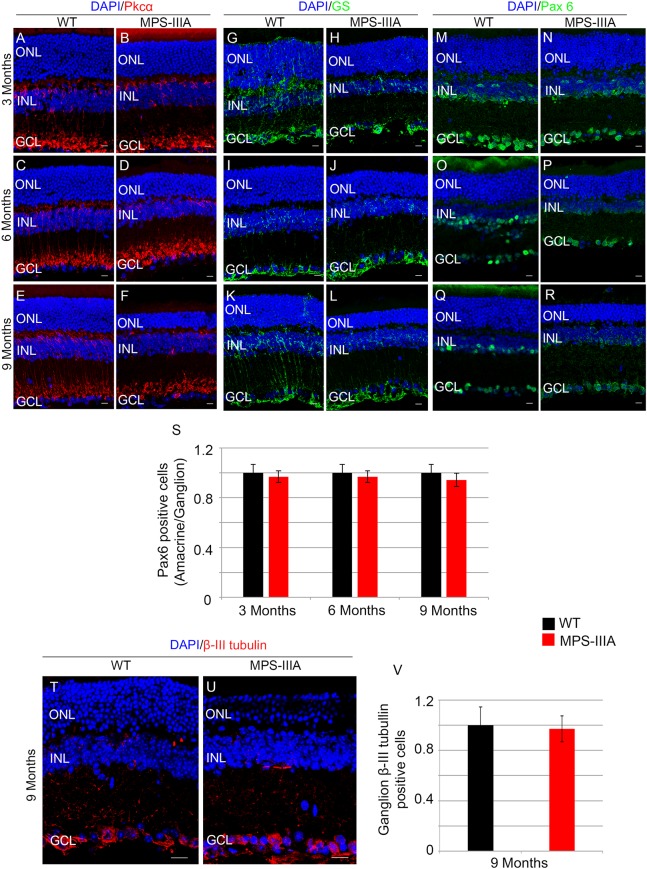
*SGSH loss of function* seems to induce a specific photoreceptor loss phenotype. Representative images of retina cryosections immunostained with anti-Pkcα **(A–F)**, anti-Glutamine Synthase (GS) **(G–L)**, and anti-Paired Box 6 (Pax 6) **(M–R)** antibodies from WT **(A,C,E,G,I,K,M,O,Q)** and MPS-IIIA **(B,D,F,H,J,L,N,P,R)** at 3 months **(A,B,G,H,M,N)**, 6 months **(C,D,I,J,O,P)**, and 9 months **(E,F,K,L,Q,R)** of age. Nuclei are counterstained with DAPI (blue). At least *n* = 6 mice per group. Scale bar 10 μm. **(S)** Graphs show the number of amacrine/ganglion Pax6-positive cells from the retina of WT and MPS-IIIA mice at 3-, 6-, and 9 months of age. Error bars represent SEM. Note that the number of Pax6-positive cells in MPS-IIIA did not change compared to WT mice. **(T,U)** Representative images of retina cryosections immunostained with anti-β-III tubulin from WT and MPS-IIIA at 9 months of age. Nuclei are counterstained with DAPI (blue). At least *n* = 6 mice per group. Scale bar 10 μm. ONL, outer nuclear layer; INL, inner nuclear layer; GCL, ganglion cell layer. **(V)** Graphs show the number of ganglion β-III tubulin-positive cells from the retina of WT and MPS-IIIA mice at 9 months of age. Error bars represent SEM. Note that the number of β-III tubulin-positive cells in MPS-IIIA did not change compared to WT mice.

**FIGURE 6:**
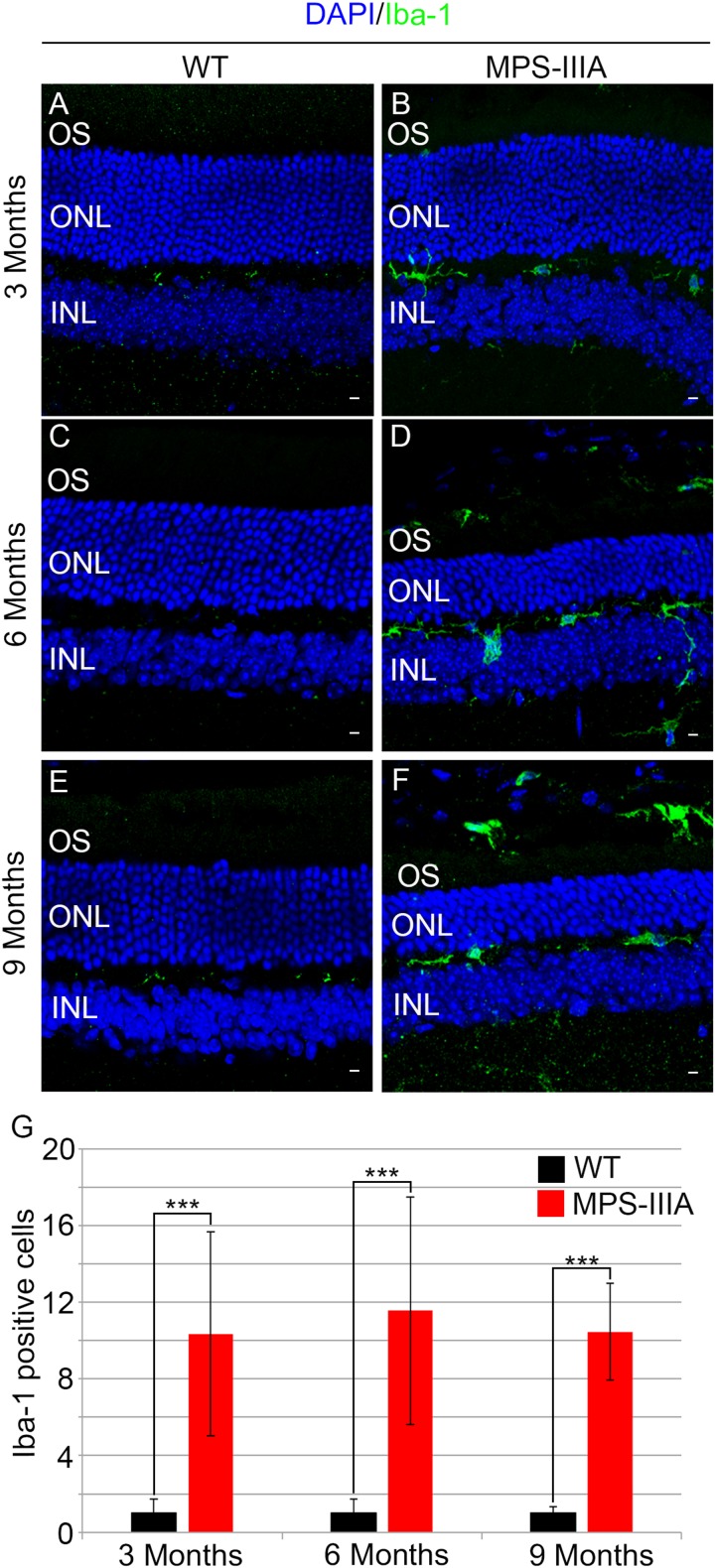
Increase of microglial activation in MPS-IIIA mouse retina. Representative images of WT **(A,C,E)** and MPS-IIIA **(B,D,F)** retinas, at 3 months **(A,B)**, 6 months **(C,D)**, and 9 months **(E,F)** of age stained with anti-Iba-1 antibody. Nuclei are counterstained with DAPI (blue). At least *n* = 6 mice per group. Scale bar 10 μm. OS, outer segment; ONL, outer nuclear layer; INL, inner nuclear layer. **(G)** Graph shows the number of Iba-1 positive cells from the retina of WT and MPS-IIIA mice at 3-, 6-, and 9 months of age. Error bars represent SEM. ***p-value ≤ 0.005 *t*-test.

To gain insight into the retinal phenotype of MPS-IIIA mice, we aimed at determining photoreceptor cell death in retinal dystrophy of MPS-IIIA mice. To that purpose, we carried out cell death assessment by TUNEL assay that revealed a significant increase in the number of apoptotic cells in the retinal ONL of MPS-IIIA mice compared to age-matched WT mice at 3-, 6-, and 9 months of age (Figures 4A–G), further supporting a photoreceptors cell death. Importantly, any significant increase in TUNEL-positive cells was detected in the other retinal layers of MPS-IIIA mice compared to age-matched WT mice. In line with these results, a detailed immunostaining analysis with markers specific for the detection of bipolar cells (PKC-α) and Müller cells (GS) showed a slight remodeling of aberrant processes of both PKC-α-bipolar and Müller positive cells in the retina of MPS-IIIA mice (Figures 5A–L), as a possible consequence of photoreceptor cell death (Marc et al., 2003). In contrast, immunostaining with anti Pax6, a marker for both amacrine and ganglion cells, did not reveal any significant defect in the number of these retinal cell types from 3- to 9 months old MPS-IIIA mice when compared with age-matched WT controls (Figures 5M–S). In accordance to these findings, the number of RGC (β-III-tubulin) in the retina of 9 months old MPS-IIIA mice did not show any significant alteration when compared with age-matched WT controls (Figures 5T–V). These results further suggest a specificity of the photoreceptor loss phenotype. However, we cannot exclude additional retinal cell dysfunction in MPS-IIIA mice that might be due to secondary storage materials and lysosomal impairment effects. Interestingly, previous studies have shown microglia activation associated to retinal dystrophy in mouse models of MPS (Karlstetter et al., 2015; Tse et al., 2015; Kruszewski et al., 2016; Reyes et al., 2017). Therefore, we asked whether the microglia was activated in the retina of MPS-IIIA mice. Immunostaining analysis on retina cryo-sections from MPS-IIIA mice with anti Iba-1, a specific marker for microglia, revealed a strong microgliosis in the retina of MPS-IIIA mice at 3-, 6-, and 9 months of age respect to age-matched WT mice (Figures 6A–G). Notably, this phenotype was also associated with a change of microglial cells localization in retinal layers. In detail, we observed that in MPS-IIIA mice the microglial cells were mainly found in the ONL and in the photoreceptor layer close to the retinal pigment epithelium (RPE), while in WT retina, they were only and rarely localized in the inner plexiform layer (IPL) or outer plexiform layer (OPL) (Figures 6A–F). In light of this, although the morphological changes of photoreceptor cells became evident at 6 months, apoptotic processes and activation of microglia were already evident at 3 months.

### Heparan Sulfate Accumulation and Autophagy Pathway Impairment: Main Players of Retinal Degeneration in MPS-IIIA Mouse Model

**FIGURE 7:**
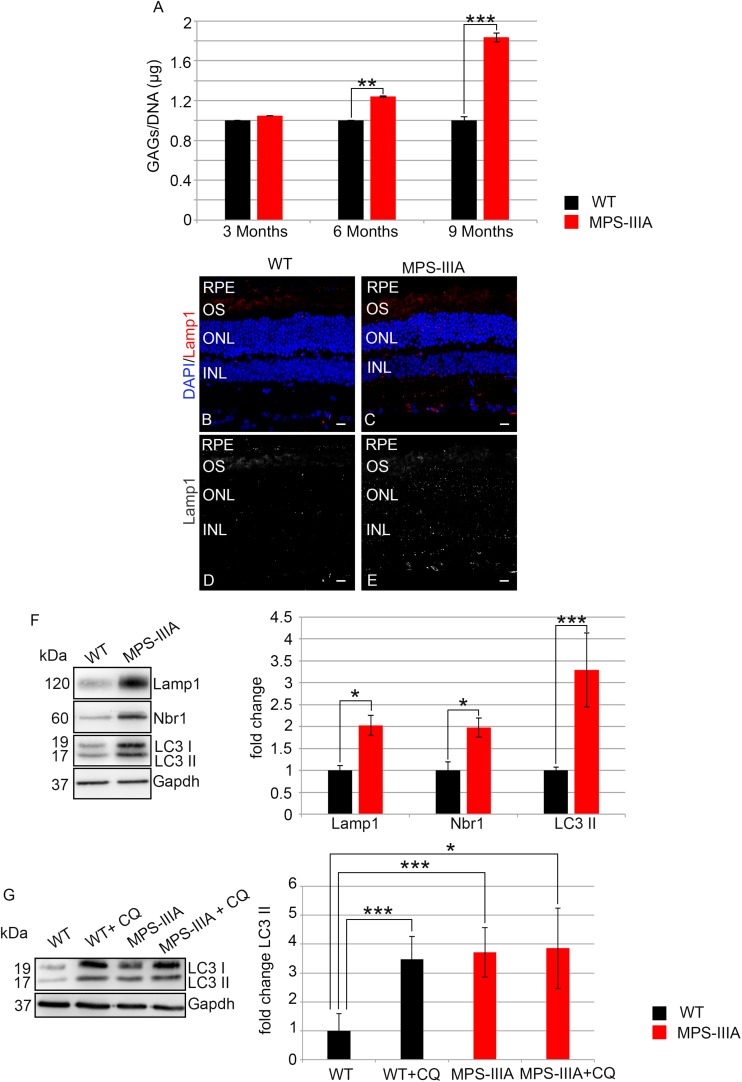
GAGs accumulation and autophagy are impaired in MPS-IIIA mouse model. **(A)** Graph shows the quantification of GAGs from the RPE of WT and MPS-IIIA mice at 3-, 6-, and 9 months of age. Error bars represent SEM. ***p-value ≤ 0.005, **p-value ≤ 0.01 *t*-test. Representative images of retina cryosections immunostained with anti-Lamp1 antibody from WT **(B,D)** and MPS-IIIA **(C,E)** mice at 9 months of age. Nuclei are counterstained with DAPI (blue). At least *n* = 6 mice per group. Scale bar 10 μm. RPE, retinal pigment epithelium; OS, outer segment; ONL, outer nuclear layer; INL, inner nuclear layer. **(F)** Representative Western blots of Lamp1, Nbr1, and LC3 proteins from WT and MPS-IIIA mice at 9 months of age. The plots show the quantification of the indicated proteins normalized to Gapdh loading control. Bar graphs represent mean values ± SEM of independent experiments (*n* = 6 mice). ***p-value ≤ 0.005, *p-value ≤ 0.05 *t*-test. **(G)** Representative Western blots of LC3 proteins from WT and MPS-IIIA mice at 9 months of age injected with chloroquine (CQ). The plots show the quantification of the LC3 II normalized to Gapdh loading control. Bar graphs represent mean values ± SEM of independent experiments (*n* = 6 mice). ***p-value ≤ 0.005, *p-value ≤ 0.05 *t*-test.

In order to assess if the photoreceptor degeneration is associated with HS storage in the same retinal compartment, we performed a quantitative measurement of GAGs in RPE of MPS-IIIA mice at different time points of retinal pathology. This analysis revealed a progressive increase of GAGs in RPE of MPS-IIIA mice from 3 to 9 months of age respect to the age-matched WT mice. In particular, at 9 months of age, we observed an increase of GAGs about twofold higher in the RPE from MPS-IIIA mice compared to age-matched WT mice (Figure 7A). Since abnormal GAGs accumulation is responsible of lysosomal compartment enlargement and autophagy impairment in CNS of MPS-IIIA mice, we evaluated if also the retinal pathology is interested by these pathological events. Immunostaining for lysosomal-associated membrane protein 1 (Lamp1) on retina cyo-sections showed an increase of Lamp1 positive cells associated with a quantitative increase of Lamp1 protein levels in the RPE of MPS-IIIA mice at 9 months of age compared to WT age-matched mice (Figures 7B–F). We next tried to understand if the RPE of MPS-IIIA mice showed together with primary storage also a block of autophagosome–lysosomal fusion. To monitor the autophagy impairment in retina of MPS-IIIA mice, we performed a quantitative analysis of LC3 protein levels in MPS-IIIA mouse retina. We observed a strong increase of lipidated form of LC3 II, associated with a significant increase of Nbr1 protein levels in 9 months old MPS-IIIA mice compared to WT age-matched mice (Figure 7F), indicating a possible block of autophagy pathway. Furthermore, to provide evidence that an impairment of autophagy occurs in the RPE of MPS-IIIA mice, as expected we demonstrated that a single acute administration of lysosomal inhibitor chloroquine (CQ) in MPS-IIIA mice at 9 months of age did not increase the levels in lipidated form of LC3 (LC3 II) compared to MPS-IIIA age-matched control mice (Figure 7G), in which the autophagy flux is already impaired. In contrast, the cloroquine treatment in the WT age-matched control mice resulted in a significant increase of LC3 II marker compared to WT control mice. Overall these data indicated that, similarly to the CNS pathology, also the retinal degeneration is associated with a progressive HS accumulation and autophagy impairment in MPS-IIIA mice.

## Discussion

The relevance of the pathological consequences associated to HS accumulation in the CNS of MPS-IIIA mouse model has been recently gathered. Nevertheless, there is only little information available on the effects of HS accumulation on retina maintenance and function. Here, we show the first evidence of a specific relationship between HS accumulation and photoreceptor function and survival in mammals. We describe a detailed physiological and morphological characterization of visual impairment in MPS-IIIA mouse model trying to outline the causes of this dysfunction. In detail, we observed a progressive decrease of retinal functionality in MPS-IIIA mice by ERG experiments at different ages 3-, 6-, and 9 months. The reduction of a-wave at early time point (3 months of age) suggests that the retinopathy is primary caused by photoreceptors degeneration. This functional deterioration of photoreceptors is associated with a progressive, significative, and specific reduction of ONL thickness of MPS-IIIA mice retina that culminates at 9 months of age in which the animals also show a severe CNS degeneration. The latter conclusion was supported by a number of evidences, a special emphasis should be placed on the lack of a TUNEL-positive cells in the inner nuclear layer (INL), even in old mice, and the unaltered interneurons morphology as assessed by immunofluorescence. Similar conditions have been described in another mouse model of Sanfilippo syndrome like MPS-IIIB and clearly observed in MPS-IIIA patients but never analyzed in MPS-IIIA mouse model (Fenzl et al., 2015; Tse et al., 2015). Since the hallmark of visual dysfunction in Sanfilippo patients and in particular in MPS-IIIA is represented by the retinopathy with pigmentary retinal degeneration associated with the loss of photoreceptors, we can affirm that our mouse model well recapitulates the functional and morphological MPS-IIIA human retinal phenotype. Another important aspect that we described in MPS-IIIA mouse model is that the retinal dystrophy is accompanied by a strong and significative inflammatory response of microglial cells (Iba-1) together with an increase of apoptotic death events in the ONL at 9 months of age. In our previous study, we observed that the neuronal death is associated with a significative activation of microglia and astroglia in the MPS-IIIA mouse brain at 9 months age, thus indicating that the CNS and retinal tissues are interested by the same cascade of inflammatory events (Sorrentino et al., 2013). Importantly, we tried to understand the causes of visual loss in MPS-IIIA mice by analyzing the impact of GAG accumulation and impairment of the autophagy flux on the retinal photoreceptor degeneration. Despite these events are well dissected for the CNS and somatic pathology, they have never been analyzed in the retina of MPS-IIIA mice. As observed in MPS-IIIA patients, the mouse model shows a significant deposition of GAGs in RPE at the early and late stage of CNS pathology. Moreover, we further observed that together with primary storage of GAGs due to the lysosomal dysfunction, the RPE layer is also characterized by autophagy impairment. Indeed, as we previously described in the CNS, the block of autophagy flux is one of the most important players of the MPS-IIIA pathology and the alteration of the LC3 autophagy marker represents a standard tool to monitor the impairment of this pathway. We demonstrated that an increase of lipidated form of LC3 (LC3 II) was clearly visible in the retina of MPS-IIIA mice at 9 months of age, indicating an impairment in autophagosome–lysosomal fusion. This phenotype was accompanied by an increase of Nbr1 protein levels further suggesting a block of ubiquitinated proteins degradation. However, impairment of the endocytic/autophagy–lysosomal pathways in the RPE has been frequently implicated in the retinal dystrophic phenotypes that are associated with severe photoreceptor cell death as described in age-related macular degeneration (AMD) (Ferrington et al., 2016; Keeling et al., 2018).

Overall these data not only confirmed the block of autophagy pathway and protein degradation impairment in the retina as observed in the CNS of MPS-IIIA mouse model, but our findings contribute in uncovering of the role of HS accumulation in photoreceptor survival and function. Finally, our results describe in the deep structural, functional, and biochemical aspects of retinal dysfunction as first pathological sign of CNS impairment in the MPS-IIIA mice highlighting the importance to carry out retinal assessment for a development of therapeutic approach for the treatment of neuropathology in MPS-IIIA disorder.

## Materials and Methods

### Animals

The MPS-IIIA mouse line (*sgsh^–/–^*) employed in this study was maintained and analyzed as described previously (Sorrentino et al., 2013). All studies on animals were conducted in strict accordance with the institutional guidelines for animal research and approved by the Italian Ministry of Health; Department of Public Health, Animal Health, Nutrition and Food Safety in accordance to the law on animal experimentation (DL 27/01/1992 No. 116). Furthermore, all animal treatments were reviewed and approved in advance by the Ethics Committee of TIGEM Institute (Pozzuoli, Italy). MPS-IIIA mice were maintained on the C57Bl/6J background and they result negative for Rd8 mutation. In all experiments, we used as control aged-matched littermates of MPS-IIIA mice. For CQ assays, WT and MPS-IIIA mice at 9 months of age underwent to single intraperitoneal injection of 120 mg/kg CQ as previously described (Soria et al., 2018) and analyzed 4 h after the injection.

### Immunofluorescence on Sections

Mouse eyes were fixed overnight in 4% paraformaldehyde in PBS at 4°C and then cryopreserved by treatment first with 15% and then with 30% sucrose in phosphate-buffered saline and embedded in OCT. Twelve-micrometer cryosections were collected on slides (Superfrost Plus; Fisher Scientific, Pittsburgh, PA, United States). The following primary antibodies were used: mouse anti-Rhodopsin (1:5000, Abcam ab3267), rabbit anti-c-Arrestin (1:1000, EMD Millipore AB15282), rabbit anti-Pax6 (1:250, Hybridoma Bank 901301), rabbit anti-PCKα (1:250, Sigma P4334), mouse anti-GS (1:200, EMD Millipore MAB302), mouse anti-β-III tubulin (1:1000, Abcam ab7751), rabbit anti-Iba-1 (1:1000, Wako 019-19741), rat anti-Lamp1 (1:400, Santa Cruz sc-19992). All incubations were performed overnight at 4°C. After washing with 1% PBS, slides were incubated with the following secondary antibodies: Alexa 594 goat anti-rat/rabbit/mouse (1:1000, Invitrogen A-11037, A-11032) or Alexa 488 goat anti-rabbit/mouse (1:1000, Invitrogen A-11008, A-11001) and DAPI (1:500, Vector Laboratories H-1200) for 1 h at room temperature, then the slides were washed with 1% PBS and mounted with PBS/glycerol or dehydrated before mounting (Eukitt Mounting Medium; EMS, Fort Washington, PA, United States). Sections were observed with a Leica DM-6000 microscope and then confocal images were acquired using the LSM710 Zeiss Confocal Microscopy system. The number of marker-positive cells was generally evaluated in the central area of the retina by manual counts with a Leica DM-6000 microscope, with the objective Leica ∞/0.17/D, HCX PL FLUOTAR, 40X/0.75 that has an area of 0.31 mm^2^.

### Western Blotting

Mouse eyes were enucleated and the RPE was separated from the retina. RPE samples were lyzed by using RIPA buffer (150 mM sodium chloride, 1% Triton X-100, 0.5% sodium deoxycholate, 0.1% sodium dodecyl sulfate, 50 mM Tris, pH 8.0) with inhibitors cocktail (Thermo Fischer Scientific, 78420). The concentration of total protein was determined by Bradford analysis and quantified by using a NanoDrop ND-8000 spectrophotometer (NanoDrop Technologies). The extracted proteins (20 μg) were fractionated by sodium dodecyl sulfate polyacrylamide gel electrophoresis (SDS-PAGE) and blotted onto a PVDF membranes (EMD Millipore, IPVH00010). After blocking with Tween 0.1%-Tris-buffered saline containing 5% non-fat milk or 5% bovine serum albumin (Sigma–Aldrich, 9048-46-8) for 1 h at room temperature, membrane filters were incubated overnight at 4°C with primary antibodies. For Western blot analysis, the following antibodies were used: anti-Lamp1 (1:500, Santa Cruz sc-19992), anti-Nbr1 (1:1000, Abnova H00004077-MO1), anti-LC3 (1:1000, Novus NB100-2220), and anti-Gapdh (1:1000, Santa Cruz sc-32233). After washing with Tween 0.1%-Tris-buffered saline, the membranes were incubated for 1 h at room temperature with the following secondary antibodies: goat anti-rabbit IgG antibody, HPR conjugate, and goat anti-mouse IgG antibody HPR conjugate (1:10,000 EMD Millipore, 12-348; 12-349). Western blot bands were revealed using a chemiluminescence digital imaging system (ImageQuant Las-4000 Mini, GE Healthcare Life Sciences) and quantified using ImageJ software.

### Electrophysiological Recordings

Scotopic and photopic electrophysiological recordings were performed as described (Barbato et al., 2017). A National Instruments amplifier with a xenon Ganzfeld stimulator (CSO, Costruzione Strumenti Oftalmici, Florence, Italy) was used to record MPS-IIIA mice. Briefly, mice were dark-adapted for 3 h, anesthetized, and positioned in a stereotaxic apparatus under dim red light. Their pupils were dilated with a drop of 0.5% tropicamide (Visufarma, Rome, Italy) and body temperature was maintained at 37.5°C. ERGs were evoked by 10 ms flashes of different light intensities ranging from 10^–4^ to 20 cd s/m^2^ generated. ERGs and b-wave thresholds were assessed using the following protocol: eyes were stimulated with light flashes increasing from −5.2 to + 1.3 log cd s/m^2^ (which corresponds to 1 × 10^–5^.^2^ to 20.0 cd s/m^2^) in scotopic conditions. The log unit interval between stimuli was 0.3 log from −5.4 to 0.0 log cd s/m^2^, and 0.6 log from 0.0 to + 1.3 log cd s/m^2^. For ERG analysis in scotopic conditions, the responses evoked by 11 stimuli (from −4 to + 1.3 log cd s/m^2^) with an interval of 0.6 log unit were considered. To minimize the noise, three ERG responses were averaged at each 0.6 log unit stimulus from −4 to 0.0 log cd s/m^2^ while one ERG response was considered for higher (0.0- + 1.3 log cd s/m^2^) stimuli. The time interval between stimuli was 10 s from −5.4 to 0.7 log cd s/m^2^, 30 s from 0.7 to + 1 log cd s/m^2^, or 120 s from + 1 to + 1.3 log cd s/m^2^. a- and b-wave amplitudes recorded in scotopic conditions were plotted as a function of increasing light intensity (from −4 to + 1.3 log cd s/m^2^). The photopic ERG was recorded after the scotopic session by stimulating the eye with 10 flashes of 10 ms with 20.0 cd s/m^2^ light intensity on a constant background illumination of 50 cd/m^2^.

### Detection of Apoptotic Cell Death

The number of apoptotic cells was analyzed by TdT-mediated dUTP nick end labeling (TUNEL), using the *In Situ* Cell Death Detection Kit, Fluorescein (Roche 11684795910) following the manufacturer’s directions. Twelve-micrometer cryosections were collected on slides and submit to TUNEL assay. To consider the presence of unspecific results, some retina sections were incubated with the reaction mix without TUNEL reaction enzyme. Sections were observed with a Leica DM-6000 microscope and then confocal images were acquired using the LSM710 Zeiss Confocal Microscopy system. The number of TUNEL-positive cells was evaluated in the central part of the retina by manual counts with a Leica DM-6000 microscope, with the objective Leica ∞/0.17/D, HCX PL FLUOTAR, 40X/0.75 that has an area of 0.31 mm^2^.

### Cone Photoreceptor Cell Counts, Assessment of ONL Density and Thickness, and OS Length Measurement

#### Cone Cell Counts

The number of cones/area was manually estimated in at least 12 retina sections immunostained with c-Arrestin from each eye sample by using a Leica DM-6000 microscope, with the objective Leica ∞/0.17/D, HCX PL FLUOTAR, 40X/0.75 that has an area of 0.31 mm^2^.

#### ONL Thickness and OS Length Measurement

At least 12 retina sections were imaged by confocal microscopy using a Leica TCS SPE 40x objective (an area of 0.31 mm^2^), using Z-stacks in the wavelength of DAPI. The ONL thickness and OS length were manually measured using ImageJ software.

For all these analyses, both central and peripheral areas of the retina sections were evaluated.

### Quantification of GAGs

To analyze GAGs in the RPE, mouse eyes were dissected to remove optic nerve, retina, cornea, and lens in ice-cold PBS 1X under stereomicroscopy (Leica). The RPE/choroid were peeled from the eyecup and transferred to a tube; 5–50 mg of RPE was dissolved in 300 μL of sterile water and treated with proteinase k 50 μg/mL at 55°C for 2 h at 750 r/min. After centrifugation at 10,000 r/min for 10 min, DNA was quantified by using Nanodrop. GAGs were characterized using Blyscan Sulfated Glycosaminoglycans Assay (Biocolor B1000) manufacturer’s protocol.

## Data Availability Statement

The datasets generated for this study are available on request to the corresponding authors.

## Ethics Statement

All studies on animals were conducted in strict accordance with the institutional guidelines for animal research and approved by the Italian Ministry of Health; Department of Public Health, Animal Health, Nutrition and Food Safety in accordance to the law on animal experimentation (DL 27/01/1992 No. 116). Furthermore, all animal treatments were reviewed and approved in advance by the Ethics Committee of TIGEM Institute (Pozzuoli, Italy).

## Author Contributions

DI, IC, and NS conceived the study, designed the experiments, and interpreted the data. AF contributed to experimental implementation and interpretation of the *in vivo* data. DI, GG, EM, VM, FS, and EN performed *in vivo* experiments. DI and GG performed the molecular biology studies and analyses on imaging data. IC and NS supervised the work and wrote the manuscript. The authors declare not to have any financial competing interests.

## Conflict of Interest

The authors declare that the research was conducted in the absence of any commercial or financial relationships that could be construed as a potential conflict of interest.
